# Our Doctors

**Published:** 1894-05-12

**Authors:** 


### Our Doctors.

(*From this week's "Punch."*)

["I think the profession, if I may presume to say so, has done well in the determination that Sir Andrew Clark might in the present age be taken up by common consent as a typical man, the representative of all that is best and noblest in the profession, and in its work."—Mr. Gladstone's speech at the meeting at Prince's Hall, on May 3rd, to further the movement for raising a memorial to the late Sir Andrew Clark.]

*Mr. Punch loquitur:—*

What the great world to its great doctors owes,
Who can in fullness tell? Who fully knows?
How many a toiler, weary and o'erworked,
In whose tired frame, all unsuspected, lurked
The incipient seeds of dire disease, or death,
Warned, soothed, relieved, will lift in grateful breath,
Whenever chance permits, warm heartfelt praise
To him, the kindly lengthener of his days—
Clark, Jenner, Gull, Mackenzie, Thompson, Roose,
Whose sympathy's softest word, whose skill's best use,
Were his at sorest need, with scant regard
To self's convenience, or to skill's reward!
Pathetic sight! Our Old Man Eloquent
Bowed down by years, and with long toil forespent,
Comes forth from well-earned rest, at much of risk,
In gratitude alert, in friendship brisk,
Though worn and weak in frame, once more to raise
That matchless voice in his great Doctor's praise.
Well-dared, well done! Well followed it *should* be.
We have not all such eloquence as he,
But we have hearts—and purses, and should all
Respond with both for that Memorial
To him, the typic doctor, and in him
Honour—it may be some with eyes half dim
With thankful recollection—all those men
Whom servants of the Sword, the Brush, the Pen,
The Forum and the Senate owe such debt
As makes us all remember with regret
We have not all the happy power to mark,
With Gladstone's eloquence, the fame of Clark.

An American contemporary points out, with much probability of truth, that the overcrowding of the medical profession is responsible in a great measure for the amount of quacks and quackery that continues to appear in our midst. The necessity of notoriety for the means of bare existence is quite probably a cause which tempts certain of the profession thus to work upon public credulity. And yet there are some parts of the globe where this plethora of medical practitioners is conspicuously absent. Take Russia for instance. Here, where the total population of the country amounts to 110,000,000, we find only 18,334 doctors registered to combat the vast numbers. This means in other words that to about every 6,000 persons there is one doctor. Practically speaking less than this exists in the actual country parts, for the sliding scale of physicians fees in Russia has driven the more mercenary away from poverty and the peasant into towns where wealthier patients can be counted upon.

